# Associations between hepatitis B virus basal core promoter/pre-core region mutations and the risk of acute-on-chronic liver failure: a meta-analysis

**DOI:** 10.1186/s12985-015-0313-5

**Published:** 2015-06-11

**Authors:** Feishu Hu, Sheng Bi, Huadong Yan, Yu Shi, Jifang Sheng

**Affiliations:** State Key Lab of Diagnostic and Treatment of Infectious Diseases, Collaborative Innovation Center for Diagnosis and Treatment of Infectious Disease, The First Affiliated Hospital of Medical School, Zhejiang University, Hangzhou, 310000 China; Department of Hepatology, Ningbo No. 2 Hospital, Ningbo, 315010 China

**Keywords:** Hepatitis B virus, Mutation, Acute-on-chronic liver failure, Hepatitis B e antigen

## Abstract

**Background:**

Several studies have suggested a relationship between hepatitis B virus (HBV) basal core promoter/pre-core mutations and HBV-induced acute-on-chronic liver failure (ACLF). Therefore, we evaluated this potential relationship using a meta-analysis.

**Methods:**

Chinese or English studies from 1966 to January 31, 2014 were included in the analysis. A random or fixed-effects model was used to merge the odds ratios (ORs).

**Results:**

We identified 31 case–control studies containing a total population of 1995 ACLF and 3822 chronic hepatitis B (CHB) patients. Several mutations were significantly correlated with ACLF: T1753V (1.889, 95 % confidence interval (CI) [1.357–2.631]), A1762T (2.696 [2.265–3.207]), G1764A (3.005 [2.077–4.347]), A1762T/G1764A (2.379 [1.519–3.727]), C1766T (1.849 [1.403–2.437]), T1768A (2.440 [1.405–3.494]), A1846T (3.163 [2.157–4.639]), G1896A (2.181 [1.800–2.642]), G1899A (3.569 [2.906–4.385]) and G1896A/A1762T/G1764A (1.575 [1.172–2.116]). Additionally, HBeAg-negative status was also statistically significant for the progression to ACLF (OR = 2.813, 95 % CI = 2.240–3.533, *p* < 0.001). However, there was no association between ACLF development and HBV genotype.

**Conclusions:**

The HBV basal core promoter/pre-core mutations T1753V, A1762T, G1764A, C1766T, T1768A, A1846T, G1896A and G1899A, and an HBeAg-negative status correlate with an increased risk of HBV-ACLF.

**Electronic supplementary material:**

The online version of this article (doi:10.1186/s12985-015-0313-5) contains supplementary material, which is available to authorized users.

## Background

Acute-on-chronic liver failure (ACLF) occurs when liver function is suddenly compromised as a result of an incident [1]. In 2009, the Asia-Pacific Association for the Study of the Liver (APASL) recommended the following definition for ACLF: acute hepatic damage showing coagulopathy and jaundice, with encephalopathy and/or ascites within 4 weeks, whether or not the patient had been diagnosed with a chronic liver disease [2]. The majority of Chinese ACLF patients showed HBV-induced hepatitis [3, 4]. Although the biological pathways that mediate HBV infection and cause liver failure remain unknown, studies suggest that mutations in the HBV viral genome may play a role in the progression of liver diseases [5–8]. HBV mutations in the basal core promoter (BCP)/pre-core (PC) regions are relatively well understood [9–11]. BCP mutations lead to enhanced HBV replication in vitro, while PC mutations break the immune tolerance of chronic HBV infection by inhibiting hepatitis B e antigen (HBeAg) translation [9–11]. Therefore, it is important to better understand the relationship between HBV mutations and ACLF, which may have clinical utility. Several studies have shown that HBV mutations in the BCP or PC regions are related to the risk of ACLF, although the sample size and the mutational sites tested were limited. Therefore, we performed a meta-analysis to quantitatively evaluate data from several available studies and investigated the relationship between HBV BCP/PC variations and the risk of developing ACLF.

## Results

### Search results

**Fig. 1:**
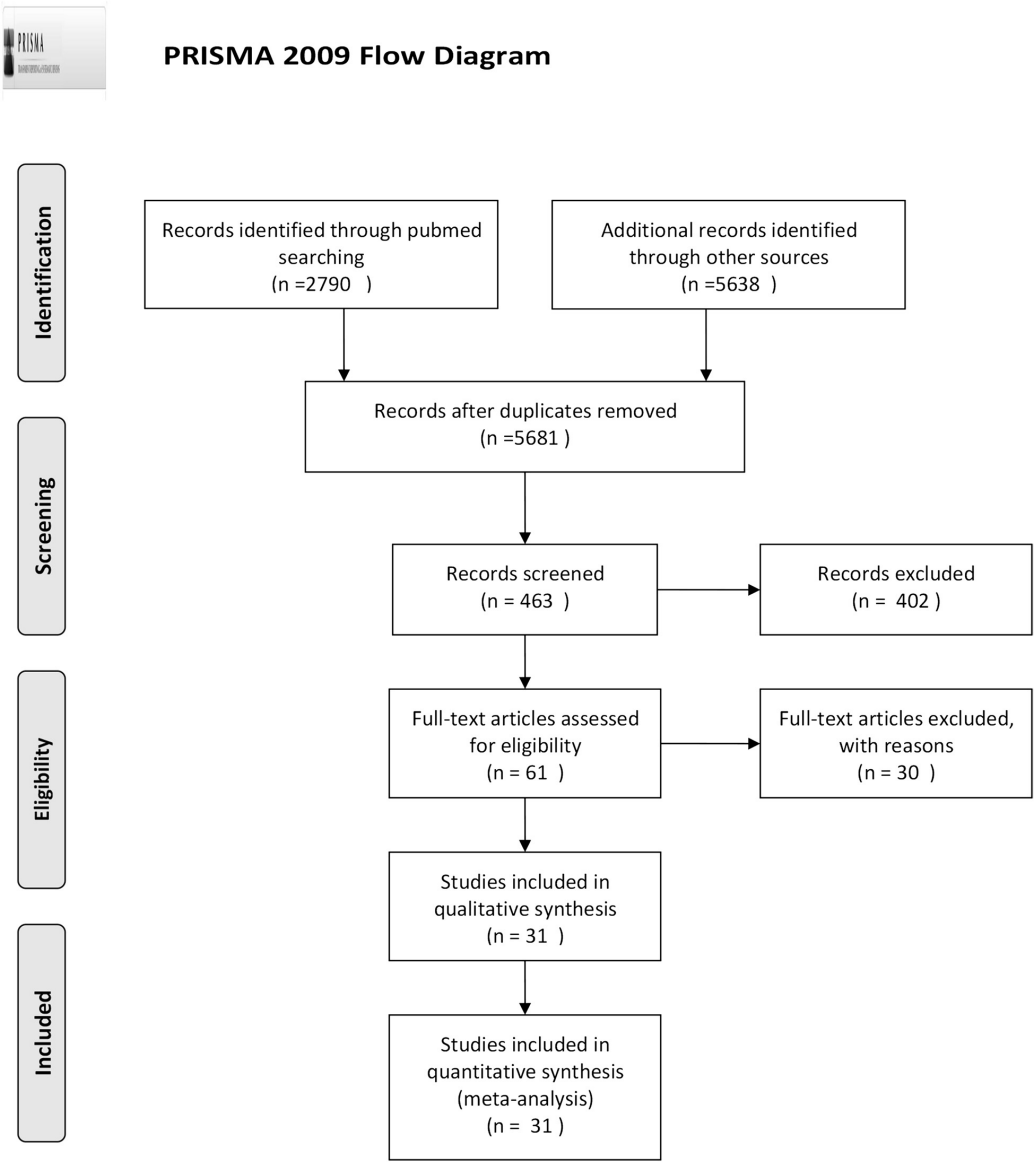
Flow chart of article selection

We identified 2790, 831, 25 and 15 articles from the PubMed, EMbase, Biomed Central and ClinicalTrials databases, respectively. The four primary Chinese databases used were: Chinese Biological Medicine, WANFANG DATA, VIP and China National Knowledge Infrastructure, which yielded 889, 1564, 1011 and 1303 studies, respectively. This approach resulted in 3478 and 2203 studies after removing duplicates from English and Chinese databases, respectively. After title screening (4908 not relevant to HBV mutation or liver failure, 310 associated with human immunodeficiency virus (HIV) or hepatitis C virus), 463 potential articles remained, which were reduced to 61 after reviewing abstracts (338 were not related to mutations, 35 not related to ACLF and 29 related to hepatocellular carcinoma [HCC]). In addition, 30 articles were removed after completing a full text scan (17 lacked control groups, 5 participants were co-infected with other hepatic viruses, 2 were co-infected with HIV and 6 had HCC). We included 31 articles in our meta-analysis (Fig. 1).

### Characteristics of trials included in this meta-analysis

The 31 trials were all case–control trials from China. The primary mutation detection method was nested polymerase chain reaction and sequencing. The quality of the studies on the Newcastle-Ottawa Scale (NOS) scale was between 5 and 7 (see Additional file 1: Table S1). There were 5817 HBV patients, 1995 of whom had ACLF. The most common mutations in the BCP/PC regions that were associated with ACLF progression were T1753V, A1762T/G1764A, A1762T, G1764A, C1766T, T1768A, A1846T, G1862T, G1896A, G1899A and G1896A/A1762T/G1764A (Table 1).

**Table 1:** Main characteristics of included trials

Study	NCA	NCO	Mutation site	Detection method	Matching factors			
					Age	Sex	Genotype	HBV DNA
Aiming Zhang, 2013	29	52	T1753V, A1762T, G1764A, A1762T/G1764A, A1846T, G1896A, G1899A	NP, AS	+	+	+	+
Aiming Zhang2, 2013	58	51	T1753V, A1762T, G1764A, A1762T/G1764A, A1846T, G1896A, G1899A	NP, AS	+	+	+	+
Lei Xiao, 2013	77	136	T1753V, A1762T/G1764A, C1766T, T1768A, A1846T, G1862T, G1896A, G1899A	NP, AS	+	+	-	-
Xiaodong Li, 2013	146	239	T1753V, A1762T/G1764A, C1766T, T1768A, A1846T, G1896A, G1899A	NP, AS	+	+	-	-
X Ren, 2010 (40)	75	328	T1753V, A1762T, G1764A, C1766T, T1768A, G1896A, G1899A	NP, AS	+	+	+	+
Zhihui Xu, 2010	298	495	T1753V, A1762T, G1764A, C1766T, T1768A, G1862T, G1896A, G1899A	NP, AS	+	+	+	-
Ling Yang, 2010	39	44	A1762T/G1764A, G1862T, G1896A, G1899A	SNP, AS	-	+	+	-
Zhengang Zhao, 2010	20	19	T1753V, A1762T, G1764A, A1762T/G1764A, C1766T, T1768A, A1846T, G1862T, G1896A, G1899A, A1762T/G1764A/G1896A	NP, DS	-	+	+	-
Xiaoyan Ma, 2012	44	28	T1753V, A1762T, G1764A, A1762T/G1764A, C1766T, T1768A, A1846T, G1896A, G1899A, A1762T/G1764A/G1896A	NP, S	-	+	+	-
Zhiwei Li, 2002	13	10	A1846T, G1862T, G1896A	RP, SA	ND	ND	ND	-
Shoubing Tang, 2005	30	30	A1762T/G1764A, G1896A	RP, CS	+	+	ND	-
Yintang Jia, 2009	8	78	T1753V, A1762T, G1764A, A1762T/G1764A, C1766T, T1768A, A1846T, G1896A, G1899A	NP, DS	ND	ND	ND	-
Ling Jiang, 2013	146	239	A1762T/G1764A, G1896A	QPMK, DS	+	+	ND	-
Wenjun Du, 2005	9	75	A1762T, G1764A, G1896A	RP, GC, S	ND	ND	ND	-
Xiumei Zhou, 2009	80	80	A1762T/G1764A, G1896A, G1899A	RP, GC	+	+	ND	-
Mingxian Zhou, 2004	22	56	A1762T, G1896A	UK	ND	ND	+	-
Xiaoqiang Ren, 2009	348	610	T1753V, A1762T, G1764A, A1762T/G1764A, C1766T, T1768A, G1862T, G1896A, G1899A, A1762T/G1764A/G1896A	NP, AS	+	+	+	-
Lei Jiang, 2010	26	78	G1896A	NP, RFLP	ND	ND	ND	-
Jinqiang Li, 2006	30	20	A1762T/G1764A, G1896A	RP, MS	+	+	ND	-
Chengyong Liu, 2007	34	186	A1762T/G1764A, G1896A, A1762T/G1764A/G1896A	RP, MGS	ND	ND	ND	-
Xinyu Liu, 2005	14	75	A1762T, G1764A, A1762T/G1764A, G1896A, A1762T/G1764A/G1896A	LAS	ND	ND	ND	-
Shuren Liang, 2003	23	68	G1896A	RP, OAS	ND	+	ND	-
Zhidong Zang, 2007	17	81	A1762T/G1764A	RP, LAS	ND	ND	ND	-
Tao Yan, 2010	49	45	T1753V, A1762T, G1764A, A1762T/G1764A, C1766T, T1768A, A1846T, G1896A, G1899A, A1762T/G1764A/G1896A	NP, AS	-	+	+	-
Wei Guo, 2006	38	40	T1753V, A1762T, G1764A, A1762T/G1764A, C1766T, T1768A, G1862T, G1896A, G1899A, A1762T/G1764A/G1896A	RP, CS	+	+	ND	-
Yanhong Yu, 2008	25	98	A1762T/G1764A	RP, GC	ND	ND	ND	-
Guanghui Wu, 2008	30	30	A1762T/G1764A, G1896A	RP, RFLP	ND	+	+	-
Hangdi Xu, 2013	12	12	A1762T, G1764A, A1846T, G1896A	RP, IS	+	+	+	+
Fan Li, 2008 (31)	87	196	T1753V, A1762T, G1764A, G1862T, G1896A, G1899A	NP, AS	ND	+	ND	-
Lu Xu, 2012	64	69	T1753V, A1762T, G1764A, A1762T/G1764A, T1768A, G1862T, G1896A, G1899A, A1762T/G1764A/G1896A	RP, AS	+	-	ND	-
Hangdi Xu2, 2013	104	254	A1846T, G1896A	SNP, AS	ND	-	-	-

*NCA* number of ACLF patients, *NCO* number of CHB patients, *S* sequencing, Apparatus unknown, *NP* nested PCR, *SNP* semi-nested PCR, *RP* routine PCR, *AS* ABI3730XL PRISM sequencing, *SA* Sequence Analysis, *CS* CEQ2000XL sequencing, *DS* direct sequencing, *QPMK* Quickchange point mutation kit, *GC* gene chip hybrid, *UK* unknown, *RFLP* restriction fragment length polymorphism, *MS* MegaBACE DNA sequencing, *MGS* Mgeabace-500 sequencing, *LAS* LicorIR2 Automatic DNA sequencing, *OAS* OpenGene Automated DNA Sequencing, *IS* Illumina sequencing, *ND* not determined, + = matched; - = unmatched; ABI PRISM 3730xl DNA Analyzer

### Patient characteristics

The studies that were included in this meta-analysis compared patients with ACLF and patients with CHB of different severities (mild, moderate and severe), or CHB patients without differentiating by severity. There were mostly males in both the ACLF and CHB groups, and the main genotypes were B and C. There were significantly fewer ACLF HBeAg-positive patients than HBeAg-negative patients, while the opposite was seen in the CHB group (Table 2).

**Table 2:** Patient characteristics of included trials

Trials	Group	N	Age, y	Gender (m/f)	Genotype (B/O)	HBeAg (P/N)	TB (μmol/L)	HBV DNA (log10IU/ml)
Aiming Zhang	A	29	43.4	27/2	7/22	11/18	330.2	3.86
	C	52	42.3	42/10	16/36	32/20	NG	4.63
Aiming Zhang2	A	58	47.4	48/10	12/46	26/32	338.4	4.02
	C	51	46.2	39/12	8/43	19/32	NG	4.69
Lei Xiao	A	77	38.2	70/7	71/6	22/55	427.5	NG
	C	136	36.9	113/23	82/54	79/57	37.62	NG
Xiaodong Li	A	146	47.4	119/27	30/116	45/101	313.4	5
	C	239	46.2	207/32	52/187	140/99	74.4	5.8
X Ren	A	75	39	67/8	23/52	29/46	451	5.3
	C	328	38	281/47	54/274	194/134	12.8	5.2
Zhihui Xu	A	298	45.9	255/43	63/235	99/199	418.5	5.48
	C-M/S	495	38.3/39.1	427/68	82/413	261/234	16.4/164.2	5.26/5.99
Ling Yang	A	39	36	36/3	24/10	11/28	353	5.75
	C	44	26	36/8	22/22	36/8	NG	9.16
Zhengang Zhao	A	20	49.7	14/6	0/16	8/12	287.37	5.82
	C	19	40.37	14/5	2/15	14/5	19.75	7.67
Xiaoyan Ma	A	44	51.6	31/13	0/44	NG	NG	NG
	C	28	38.7	20/8	2/26	NG	NG	NG
Zhiwei Li	A	13	21 to 58	17/6	NG	NG	NG	NG
	C	10			NG	NG	NG	NG
Shoubing Tang	A	30	21	25/5	NG	13/17	NG	1.47
	C	30	20	24/6	NG	16/14	NG	3.29
Yintang Jia	A	8	NG	NG	NG	NG	NG	NG
	C	78	NG	NG	NG	NG	NG	NG
Ling Jiang	A	146	46	119/27	NG	49/97	313.4	5
	C-M/S	239	41/38	206/33	NG	146/93	42.38/106.54	6.27/5.39
Wenjun Du	A	9	NG	NG	NG	NG	NG	NG
	C	75	NG	NG	NG	NG	NG	NG
Xiumei Zhou	A	80	48.6	108/72	NG	NG	NG	NG
	C	80			NG	NG	NG	NG
Mingxian Zhou	A	22	NG	NG	3/19	NG	NG	4.31
	C	56	NG	NG	9/47	NG	NG	4.72
Xiaoqiang Ren	A	348	46	292/56	53/200	132/216	255.9	4.55
	C-M/S	610	38/35	518/92	95/476	386/224	12.4/55.4	4.43/5.21
Lei Jiang	A	26	NG	NG	NG	NG	NG	NG
	C	78	NG	NG	NG	NG	NG	NG
Jinqiang Li	A	30	48.1	24/6	NG	NG	NG	NG
	C	20	43.9	15/5	NG	NG	NG	NG
Chengyong Liu	A	34	NG	NG	NG	NG	NG	NG
	C	186	NG	NG	NG	NG	NG	NG
Xinyu Liu	A	14	NG	NG	NG	NG	NG	NG
	C	75	NG	NG	NG	NG	NG	NG
Shuren Liang	A	23	39.71	18/5	NG	NG	NG	NG
	C-M0/M1/S	68	35/36.1/35.5	55/13	NG	NG	NG	NG
Zhidong Zang	A	17	NG	10/7	NG	NG	342.3	2.25
	C	81	NG	64/17	NG	NG	51.9	1.83
Tao Yan	A	49	45.8	42/7	9/40	23/26	312.2	4.88
	C	45	33.9	36/9	12/33	36/9	16.5	6.91
Wei Guo	A	38	32.6	34/4	NG	21/17	NG	5.09
	C	40	30.2	33/7	NG	26/14	NG	5.48
Yanhong Yu	A	25	NG	NG	NG	NG	NG	NG
	C	98	NG	NG	NG	NG	NG	NG
Guanghui Wu	A	30	40	25/5	6/24	NG	NG	NG
	C	30	29	20/10	4/26	NG	NG	NG
Hangdi Xu	A	12	35	11/1	10/2	4/8	324.9	6.71
	C	12	35.7	10/2	8/4	10/2	15.6	6.74
Fan Li	A	87	45.4	69/18	NG	27/60	331	4.42
	C	196	38.8	170/26	NG	130/66	23.8	5.42
Lu Xu	A	64	35.1	57/7	NG	31/33	372.2	3.89
	C	69	29.5	48/21	NG	53/16	46.5	5.49
Hangdi Xu2	A	104	40.9	91/13	68/36	51/53	317.5	6.09
	C-M/S	254	37.6/40.7	186/68	51/203	185/69	14.9/98.3	6.14/5.77

*A* ACLF patient group, *C* CHB patient group, *N* number of subjects, *M0* mild, *M1* moderate, *S* severe, *NG* not given, *m/f* male/female, *B/O* genotype B/other genotypes, *TB* Total bilirubin, *HBeAg* hepatitis B e antigen, *P* HBeAg positive, *N* HBeAg negative, *PTA* Prothrombin activity, *ALT* alanine aminotransferase, *AST* Aspertate Aminotransferase

### Data synthesis

#### Overall risk estimates

**Fig. 2:**
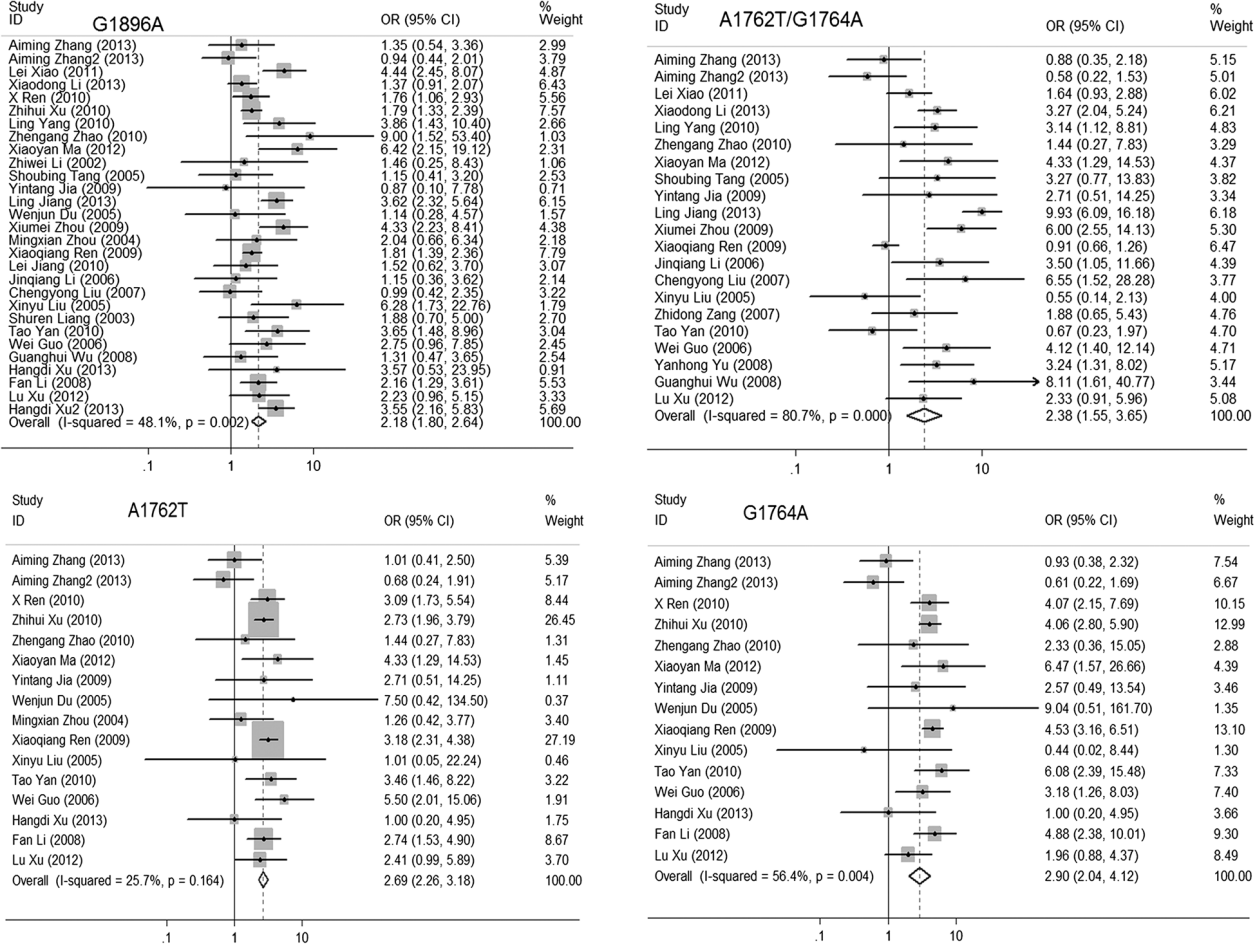
Summary odds ratio (OR) of acute-on-chronic liver failure for A1762T, G1764A, A1762T/G1764A and G1896A mutations. *Squares* represent study-specific estimates (size of the square reflects the study-specific statistical weight); *horizontal lines* represent 95 % confidence intervals (CIs); *diamonds* represent summary estimates with corresponding 95 % CIs. Test for heterogeneity: (G1896A) *p* = 0.002, I^2^ = 48.1 %; (A1762T/G1764A) *p* < 0.001, I^2^ = 80.7 %; (A1762T) *p* = 0.164, I^2^ = 25.7 %; (G1764A) *p* = 0.004, I^2^ = 56.4 %. A random-effects model was used for G1896A, A1762T/G1764A and G1764A, and a fixed-effects model was used for A1762T. All statistical tests were two-sided

The risk assessment for ACLF patients with the mutations of interest in the individual trials is summarized in Additional file 2: Table S2 and Fig. 2. Overall, the following mutations were found to be meaningfully related to ACLF risk (shown as summary OR and 95 % [CI]): T1753V (1.919 [1.414–2.606]), A1762T (2.685 [2.264–3.185]), G1764A (2.901 [2.041–4.122]), A1762T/G1764A double mutation (2.376 [1.548–3.648]), C1766T (1.849 [1.403–2.437]), T1768A (2.199 [1.563–3.094]), A1846T (3.163 [2.157–4.639]), G1896A (2.181 [1.800–2.642]), G1899A (3.525 [2.882–4.312]) and G1896A/A1762T/G1764A triple mutation (1.575 [1.172–2.116]). G1862T was not significantly associated with ACLF risk, but when we removed the study conducted by Xiaoqiang Ren, statistical significance was achieved (summary OR = 2.579 [1.510–4.405], *p* = 0.001).

#### Subgroup analysis

We evaluated two subgroups, one matched by only patient age and sex, and the other matched by HBV genotype and DNA load, age and sex. The summary OR of ACLF risk for several variations was lower in matched subgroups than that in unmatched subgroups, such as T1753V, A1762T, G1764A, C1766T, G1896A, G1899A and A1846T, while an opposite result was observed for A1762T/G1764A and T1768A (Table 3).

**Table 3:** Pooled unadjusted risk estimates of ACLF after stratification or matching

Mutation site	Matching factor	OR	_LCI	_UCI	*P*
T1753V	unmatched	1.919	1.414	2.606	0.000
	age and sex matched	1.481	0.982	2.234	0.061
	age, sex, genotype and HBV DNA matched	ND	ND	ND	ND
A1762T	unmatched	2.685	2.264	3.185	0.000
	age and sex matched	2.307	1.555	3.422	0.000
	age, sex, genotype and HBV DNA matched	1.326	0.585	3.007	0.499
G1764A	unmatched	2.901	2.041	4.122	0.000
	age and sex matched	2.023	1.013	4.041	0.046
	age, sex, genotype and HBV DNA matched	1.310	0.555	3.094	0.538
A1762T/G1764A	unmatched	2.376	1.548	3.648	0.000
	age and sex matched	2.602	1.270	5.333	0.009
	age, sex, genotype and HBV DNA matched	ND	ND	ND	ND
G1896A	unmatched	2.181	1.800	2.642	0.000
	age and sex matched	2.014	1.505	2.695	0.000
	age, sex, genotype and HBV DNA matched	1.479	1.017	2.152	0.041
G1899A	unmatched	3.525	2.882	4.312	0.000
	age and sex matched	3.171	2.464	4.081	0.000
	age, sex, genotype and HBV DNA matched	ND	ND	ND	ND
C1766T	unmatched	1.849	1.403	2.437	0.000
	age and sex matched	1.719	1.226	2.411	0.002
	age, sex, genotype and HBV DNA matched	ND	ND	ND	ND
T1768A	unmatched	2.199	1.563	3.094	0.000
	age and sex matched	2.203	1.425	3.405	0.000
	age, sex, genotype and HBV DNA matched	ND	ND	ND	ND
A1846T	unmatched	3.163	2.157	4.639	0.000
	age and sex matched	2.854	2.127	3.831	0.000
	age, sex, genotype and HBV DNA matched	ND	ND	ND	ND

*ND* not determined, *OR* odds ratio, *_LCI* low limit of 95 % confidence interval, *_UCI* upper limit of 95 % confidence interval

#### Prediction of ACLF by HBV mutation

We evaluated mutation frequency as a possible biomarker to predict ACLF. Most of the mutation sites showed low sensitivity and relatively high specificity, except A1762T, which had a sensitivity of 74.5 % (95 % CI = 71.9–76.9 %) and specificity of 45.8 % (95 % CI = 43.7–47.9 %). G1764A showed a high positive prediction value (PPV; 80.1 %, 95 % CI, 77.7–82.4; Table 4).

**Table 4:** Sensitivity and specificity of single and combined mutations for prediction of ACLF

Mutation site	Mutation status	No. of ACLF patients	No. of patients without ACLF	Sensitivity % (95 % CI)	Specificity, % (95 % CI)	PPV	NPV
T1753V	pos	392	397	29.3 (26.9 to 31.9)	83.3 (81.8 to 84.8)	49.7 (46.1 to 53.2)	67.8 (66.0 to 69.5)
	neg	945	1986				
A1762T	pos	872	1207	74.5 (71.9 to 76.9)	45.8 (43.7 to 47.9)	41.9 (39.8 to 44.1)	77.3 (75.0 to 80.0)
	neg	299	1019				
G1764A	pos	921	228	43.0 (40.9 to 45.1)	80.6 (78.3 to 82.9)	80.1 (77.7 to 82.4)	43.7 (41.6 to 45.9)
	neg	1221	949				
A1762T/G1764A	pos	678	825	51.6 (48.9 to 54.4)	63.1 (61.1 to 65.1)	45.1 (42.6 to 47.7)	69.0 (66.9 to 71.0)
	neg	635	1412				
C1766T	pos	113	118	10.3 (8.6 to 12.2)	94.1 (93.0 to 95.1)	48.9 (42.3 to 55.6)	65.8 (64.0 to 67.5)
	neg	986	1897				
T1768A	pos	80	64	6.9 (5.5 to 8.5)	96.9 (96.1 to 97.6)	55.6 (47.1 to 63.8)	65.1 (63.4 to 66.8)
	neg	1083	2020				
A1846T	pos	304	251	54.7 (50.4 to 58.9)	72.8 (69.8 to 75.6)	54.8 (50.5 to 59.0)	72.7 (69.7 to 75.5)
	neg	252	670				
G1896A	pos	1025	1254	52.6 (50.4 to 54.8)	65.6 (64.0 to 67.1)	45.0 (42.9 to 47.0)	72.1 (70.5 to 73.6)
	neg	924	2386				
G1899A	pos	323	182	22.2 (20.1 to 24.4)	92.7 (91.7 to 93.7)	64.0 (59.6 to 68.2)	67.2 (65.6 to 68.8)
	neg	1133	2325				
G1896A/A1762T/G1764A	pos	107	135	17.6 (14.7 to 20.9)	87.4 (85.2 to 89.3)	44.2 (37.9 to 50.7)	65.1 (62.6 to 67.6)
	neg	500	934				

*Pos* mutation positive, *neg* mutation negative, *PPV* positive predictive value, *NPV* negative predictive value

#### HBV genotype and HBeAg status

**Fig. 3:**
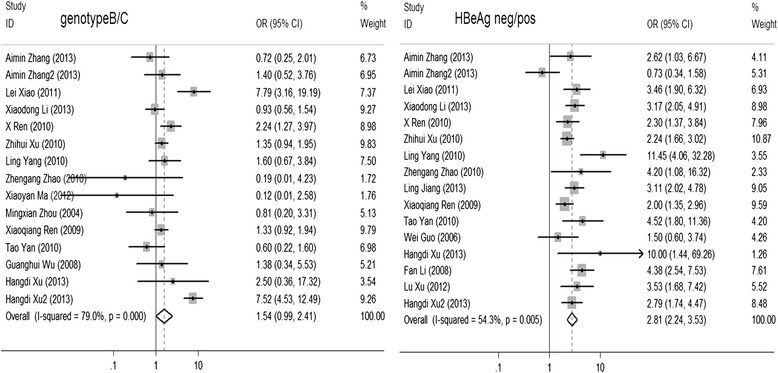
Summary OR of acute-on-chronic liver failure for genotypes and HBeAg status. Summary OR for genotypes B and C and for HBeAg-negative to HBeAg-positive status. *Squares* represent study-specific estimates (the size of the square reflects the study-specific statistical weight); *horizontal lines* represent 95 % CIs; *diamonds* represent summary estimates with corresponding 95 % CIs. Test for heterogeneity: *p* < 0.001, I^2^ = 79.0 %. A random-effects model was used. All statistical tests were two-sided

The summary OR of HBeAg-negative was 2.813 (95 % CI [2.240–3.533], *p* < 0.001). However, there was no evidence indicating that the HBV genotype B was significantly related to ACLF (Fig. 3), although we found that this result was unstable using a sensitivity analysis of the following studies: Aiming Zhang, Xiaodong Li, Zhengang Zhao, Xiaoyan Ma, Mingxian Zhou and Tao Yan. When these studies were removed, the summary OR p value was less than 0.05, indicating that more studies were needed to determine the relationship between HBV genotypes and ACLF development.

### Assessment of within-group heterogeneity, publication bias and sensitivity analysis

**Fig. 4:**
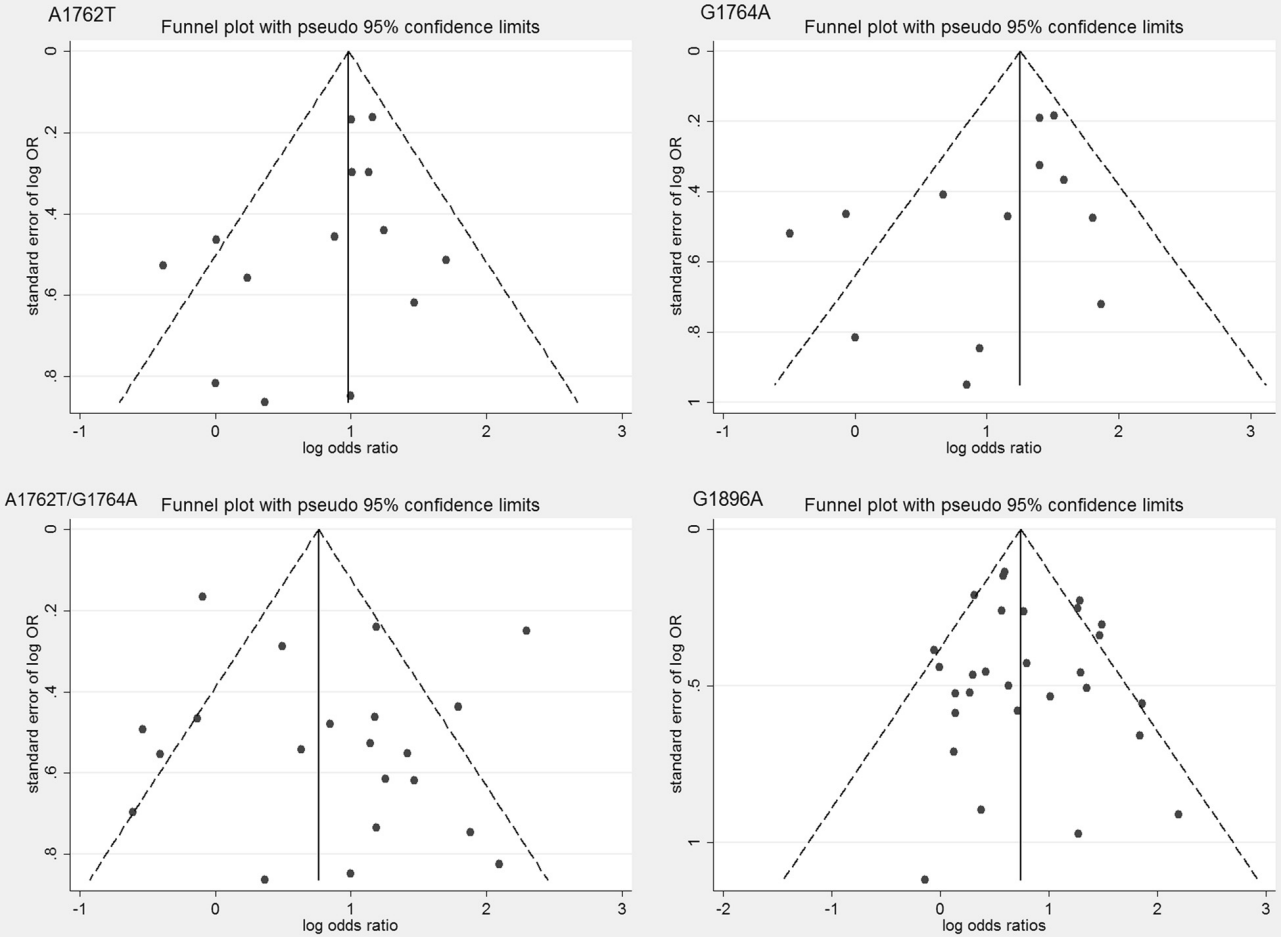
Funnel plot for A1762T, G1764A, A1762T/G1764A and G1896A. The *dashed line* represents 95 % CIs. *Circles* represent individual studies

There was a large amount of heterogeneity between different trials for T1753V, G1764A, A1846T, G1862T, G1896A and A1762T/G1764A. We therefore conducted a sensitivity analysis using a “leave one out” approach and Egger’s testing, and found that these mutations were not influential, except for G1862T. Except for those with G1764A and G1896A/A1762T/G1764A, we did not observe any publication bias with T1753V, A1762T, A1762T/G1764A, C1766T, T1768A, A1846T, G1862T, G1896A or G1899A, (Table 5 and Fig. 4).

**Table 5:** Assessment of within-group heterogeneity, publication bias and sensitivity analysis

Mutation site	*P* value for heterogeneity	*P* value for Egger’s test	Sensitivity analysis
T1753V	0.006	0.923	-
A1762T	0.164	0.181	-
G1764A	0.004	0.100	-
A1762T/G1764A	0.000	0.455	-
C1766T	0.852	0.300	-
T1768A	0.681	0.291	-
A1846T	0.037	0.856	-
G1862T	0.001	0.125	+
G1896A	0.002	0.520	-
G1899A	0.307	0.591	-
G1896A/A1762T/G1764A	0.073	0.045	-

The summary OR did not change statistical significance when running sensitivity analysis. ‘+’ = the *P* value for summary OR reached statistical significance of 0.001 when the trial conducted by Xiaoqiang Ren was removed

## Discussion

Our meta-analysis showed that T1753V, A1762T, G1764A, A1762T/G1764A, C1766T, T1768A, A1846T, G1896A, G1899A and G1896A/A1762T/G1764A were all related to increasing the risk of HBV-ACLF by a factor of 1.919, 2.685, 2.901, 2.376, 1.849, 2.199, 3.163, 2.181, 3.525 and 1.575, respectively.

To further confirm these results, several significant confounders were matched. Age and sex have been shown to influence progression of high-level hepatic diseases in CHB patients [12]. A high HBV DNA copy number may independently accelerate progression of hepatocirrhosis and HCC [13, 14]. Additionally, compared with genotype B, genotype C was related to more aggressive hepatic necroinflammation, a more rapid rate of progression to cirrhosis and HCC development [15–17], although an association between HBV genotypes B and C and ACLF was not suggested in our study. Most mutations remained significantly associated with ACLF development in age and sex-matched groups and the association with G1896A was still significant after matching age, sex, HBV genotype and HBV DNA load. However, the summary ORs were higher in the confounder-unmatched and age- or sex-only-matched studies than in the age, sex, genotype and DNA load-matched studies. Thus, our results suggest a significant association between HBV variations in BCP/PC regions and the risks of developing ACLF.

The causal relationship between HBV BCP/PC mutations and ACLF development remains unknown. ACLF development was promoted by both acute factors (precipitating events) and chronic factors (underlying chronic liver diseases) [18]. Previous studies indicated that the role of HBV BCP/PC mutations that promote ACLF were more likely to influence the severity of chronic factors, because the mutations were detected both in CHB and HBV-ACLF patients. It was also reported that HBV BCP/PC variations appeared less frequently in CHB patients than in cirrhotic patients [19, 20], suggesting a process where mutations accumulate during CHB infection and are associated with late-stage viral infection. Thus, these results suggested that patients with BCP/PC mutations had more advanced HBV-related chronic liver diseases, and thus were more susceptible to hepatic insult and more likely to develop ACLF. Accumulating evidence suggests that mutations in the BCP/PC region might exacerbate liver injury by affecting HBeAg expression and/or viral replication, which may in turn affect the immune response to HBV [21]. For example, Yang et al. suggested that HBeAg expression in the perinatal infection period caused immune tolerance [22]. Another characteristic of HBeAg that might accelerate chronic infection is that it may imitate the core protein to weaken the immune response caused by antibodies in infected hepatocytes [10]. When the occurrence of HBV mutants in different phenotypes disturbs the balance, the altered virus–cell relationship might activate adverse immune responses in some patients, causing massive hepatocyte necrosis.

When exploring the potential value of HBV variation in predicting HBV-ACLF, we found that T1753V, G1764A, C1766T, T1768A, G1899A and G1896A/A1762T/G1764A triple mutations were useful in predicting ACLF. A combination of A1762T/G1764A and G1896A showed a higher specificity than A1762T/G1764A double mutations (Table 4), suggesting that mutations that accumulate at different sites have synergistic effects in promoting the risk of HBV-ACLF. The results indicated that the above HBV mutations may be used as biomarkers for supporting the development of HBV-associated ACLF.

Our study also showed the relationship between HBeAg-negative status and the risk of ACLF using a pooled OR = 2.813, *p* < 0.001. However this relationship was probably not independent. In G1896A patients, the concentration of serum toll-like receptor 2 (TLR2) reached a significantly higher level than in HBV-infected patients lacking G1896A [23]. In the absence of HBeAg, HBV up-regulated TLR2 expression on CD14+ cells [24]. TLR2 is a potential connection between G1896A and HBeAg-negative status.

This study had several limitations. First, all trials were observational so that unidentified confounders could not be eliminated. Second, because we searched only Chinese and English articles, selection bias based on language could not be avoided. Third, the data was insufficient to allow analysis of mutations such as G1896A/G1899A, G1896A/G1899A/A1762T/G1764A and C1913V that were implicated in ACLF development in some studies [25–31]. Additionally, HBV mutations in other regions were not analyzed in the study. For example, pre-S/S region mutations were observed at an advanced stage of chronic HBV infection [32, 33]. The pre-S deletion mutation has been reported to increase with liver disease progression [34]. In addition, pre-S/S region mutations could alter the immune response against HBV by affecting neutralization antibody epitopes [35] or cytotoxic T lymphocyte epitope binding affinity [36]. Thus, ongoing studies are needed to evaluate the clinical relevance of rare mutations in the BCP region and mutations in other regions. Fourth, the conclusions in this article might not be universally applicable, because all the selected trials were conducted in China. Although no study has reported a direct relationship between the risk of ACLF and country or race, the distribution of HBV genotypes or quasispecies differs among countries and races. For example, the most prevalent HBV genotypes in China are B and C, whereas genotypes A and D are the most prevalent in Europe [37]. Although our analysis did not show a different association between genotype B or C and ACLF risk, it is unknown whether there was a different connection between other HBV genotypes and ACLF. Thus, this conclusion needs to be validated for countries or races where other HBV genotypes are the most prevalent. Fifth, summary ORs were not conducted in most manuscripts included in this meta-analysis and there were no adjusted ORs. Sixth, if the sample size were sufficiently large, grouping the HBV patients based on grades, such as HBV carriers, CHB and hepatocirrhosis, would show whether the mutations accumulated during CHB progressed to advanced liver diseases, including ACLF.

We discovered that HBV BCP/PC variations such as T1753V, A1762T, G1764A, A1762T/G1764A, C1766T, T1768A, A1846T, G1896A, G1899A and G1896A/A1762T/G1764A, and HBeAg-negative status correlated with an increased risk of HBV-ACLF. Detecting these variations in CHB patients may help to identify those who are at a high risk of developing ACLF. However, further prospective studies are needed to confirm our findings and whether HBV BCP/PC mutations contribute directly to the development of ACLF. The effect of HBV BCP/PC mutations on host viral-specific immunity also needs to be clarified.

## Conclusions

The HBV BCP/PC mutations T1753V, A1762T, G1764A, C1766T, T1768A, A1846T, G1896A and G1899A, and an HBeAg-negative status correlate with an increased risk of HBV-ACLF.

## Methods

### Search strategy and inclusion/exclusion criteria

We searched PubMed using the following strategy: (“Hepatitis B virus” [Mesh]) AND “mutation” [Mesh] from January 1, 1950 to January 31, 2014. The search strategy for EMbase was “pub-date > 1994 and TITLE-ABSTR-KEY (hepatitis B virus), and TITLE-ABSTR-KEY (mutation)”. Biomed Central was searched using “hepatitis B virus (All words) in Title + Abstract + Text, and mutation (All words) in Title + Abstract + Text from 1997 to 2014”. ClinicalTrials was searched with “hepatitis B virus” AND “mutation” without limitation. The main Chinese databases were searched using: Chinese Biological Medicine (1978 to January 31, 2014), WANFANG DATA (1997 to January 31, 2014), VIP (1989 to January 31, 2014), China National Knowledge Infrastructure (1994 to January 31, 2014); the search strategy was “hepatitis B” [Abstract] AND “mutation” [Abstract]. The inclusion criteria were: 1) studies contained control groups; 2) ACLF diagnosis was based on the APASL criteria, as follows: serum bilirubin ≥5 mg/dl and coagulopathy (INR ≥ 1.5 or PTA < 40 %) are mandatory. Ascites and/or encephalopathy were determined by physical examination within 4 weeks [38], or the ACLF diagnosis was based on the criteria recommended by the Chinese Society of Infectious Disease and the Chinese Society of Hepatology: HBV infection history; total serum bilirubin >10 mg/dl, PTA < 40 % and recent complications that also meet APASL criteria; 3) BCP or PC mutations were detected; 4) the outcome was ACLF; and 5) ORs and 95 % CIs were shown or could be calculated from original data. Studies without control groups or studies in which patients were co-infected with another hepatitis virus or HIV, with the presence of other underlying chronic liver diseases (such as Wilson disease and autoimmune hepatitis) or liver cancer were excluded. For studies that contained over-lapping populations, the study containing the larger number of participants or the most recent study was included.

### Data extraction

Feishu Hu and Sheng Bi extracted information independently as follows: publication year, location, study design, number and subject characteristics, HBV mutations, genotypes, HBeAg status, variation testing approach and probable confounding factors. All authors discussed results and reached an agreement if there were discrepancies. BCP/PC region mutation sites were included. Patients co-infected with wild-type viruses and BCP/PC mutated viruses were excluded. Subjects with a single mutation were excluded when assessing A1762T/G1764A double mutations and A1762T/G1764A/G1896A triple mutations.

### Study quality assessment

Feishu Hu and Sheng Bi independently evaluated the quality of trials using the NOS, which was developed for case–control studies. Each study that was included was judged on three broad perspectives: selection of study groups, comparability of groups and ascertainment of exposure for case–control studies. Disagreements were resolved by discussion between all authors.

### Statistical analysis

The effect measures were ORs and the corresponding 95 % CIs. Within-study heterogeneity was assessed using Cochrane’s Q-test and the I^2^ test [39]. Summary ORs were pooled by a random or fixed-effect model based on the results of a heterogeneity test. To detect the stability with primary analysis, we conducted a sensitivity analysis using a “leave one out analysis”. Publication bias was detected using funnel plots and the Egger test [40]. Significant publication bias was believed to be present if a p value was less than 0.1. We also conducted subgroup analysis by stratifying the study participants according to probable confounders such as sex, age, HBV DNA and HBV genotype and we calculated the respective summary risk estimates. We used Stata software (version 12.0; Stata Corp, College Station, TX) to conduct the analysis and to calculate the ACLF summary estimates for the HBV genotype, HBeAg status and T1753V, A1762T, G1764A, A1762T/G1764A, C1766T, T1768A, A1846T, G1862T, G1896A, G1899A and A1762T/G1764A/G1896A mutations for ACLF patients. The following mutation sites were also detected in the included studies: G1613A, C1653T, 1752G, T1754V, T1753V/A1762T/G1764A, T1758C, G1764A/C1766T/T1768A, T1770A, 1773 T, G1775A, C1799V, T1800C, T1803C, G1809T, A1814C, A1837G, A1846G, T1853C, T1858C, G1896A/G1899A, G1896A/G1899A/A1762T/G1764A, C1913V, 1915A/C, T1938C, A1979G, T1961V and T1753V/A1762T/G1764A/G1896A/G1899A, but they were not included in this meta-analysis because the data was insufficient. Variation frequency between CHB patients and HBV-ACLF patients was compared using the Chi-squared test. Sensitivity and specificity of HBV mutations for ACLF was evaluated to indicate the possibility of using certain mutations to predict the development of HBV-ACLF.

## Additional files

Additional file 1: Table S1. The Newcastle-Ottawa Scale (NOS) for assessing the quality of the included studies. Additional file 2: Table S2. Pooled unadjusted risk estimates of ACLF of specific mutation sites.
