# Supplementary material for: Associations between hepatitis B virus basal core promoter/pre-core region mutations and the risk of acute-on-chronic liver failure: a meta-analysis
Source: Virol J. 2015 Jun 11;12:87. doi: 10.1186/s12985-015-0313-5 (PMC4485863; doi:10.1186/s12985-015-0313-5)
Supplement: Additional file 1: Table S1. — The Newcastle-Ottawa Scale (NOS) for assessing the quality of the included studies. [file 12985_2015_313_MOESM1_ESM.doc]

**Additional file 1: Table S1. The Newcastle-Ottawa Scale (NOS) for assessing the quality of the included studies**

| Study(first author) | Selection | Comparability | Exposure | Total score |
| --- | --- | --- | --- | --- |
| Aiming Zhang | ☆☆☆ | ☆☆ | ☆☆ | 7 |
| Lei Xiao | ☆☆☆ | ☆ | ☆☆ | 6 |
| Xiaodong Li | ☆☆☆ | ☆ | ☆☆ | 6 |
| X Ren | ☆☆☆ | ☆ | ☆☆ | 6 |
| Zhihui Xu | ☆☆☆ | ☆☆ | ☆☆ | 7 |
| Ling Yang | ☆☆☆ | ☆ | ☆☆ | 6 |
| Zhengang Zhao | ☆☆☆ | ☆ | ☆☆ | 6 |
| Xiaoyan Ma | ☆☆☆ | ☆ | ☆☆ | 6 |
| Zhiwei Li | ☆☆☆ |  | ☆☆ | 5 |
| Shoubing Tang | ☆☆☆ | ☆☆ | ☆☆ | 7 |
| Yintang Jia | ☆☆☆ |  | ☆☆ | 5 |
| Ling Jiang | ☆☆☆ | ☆☆ | ☆☆ | 7 |
| Wenjun Du | ☆☆☆ |  | ☆☆ | 5 |
| Xiumei Zhou | ☆☆☆ | ☆☆ | ☆☆ | 7 |
| Mingxian Zhou | ☆☆☆ |  | ☆☆ | 5 |
| Xiaoqiang Ren | ☆☆☆ | ☆☆ | ☆☆ | 7 |
| Lei Jiang | ☆☆☆ |  | ☆☆ | 5 |
| Jinqiang Li | ☆☆☆ | ☆☆ | ☆☆ | 7 |
| Chengyong Liu | ☆☆☆ |  | ☆☆ | 5 |
| Xinyu Liu | ☆☆☆ |  | ☆☆ | 5 |
| Shuren Liang | ☆☆☆ | ☆ | ☆☆ | 6 |
| Zhidong Zang | ☆☆☆ |  | ☆☆ | 5 |
| Tao Yan | ☆☆☆ | ☆ | ☆☆ | 6 |
| Wei Guo | ☆☆☆ | ☆☆ | ☆☆ | 7 |
| Yanhong Yu | ☆☆☆ |  | ☆☆ | 5 |
| Guanghui Wu | ☆☆☆ | ☆ | ☆☆ | 6 |
| Hangdi Xu | ☆☆☆ | ☆☆ | ☆☆ | 7 |
| Fan Li | ☆☆☆ | ☆ | ☆☆ | 6 |
| Lu Xu | ☆☆☆ | ☆ | ☆☆ | 6 |
| Hangdi Xu2 | ☆☆☆ | ☆ | ☆☆ | 6 |

The selection contains four aspects:1) Is the case definition adequate? 2) Representativeness of the cases. 3) Selection of Controls. 4)definition of controls. Every question worths one ☆. One ☆ is for the most important factor and a second important factor controlling respectively. Exposure includes three questions:1) Ascertainment of exposure 2) Same method of ascertainment for cases and controls 3) Non-Response rate. Every aspect is for one ☆
