# Supplementary material for: Associations between hepatitis B virus basal core promoter/pre-core region mutations and the risk of acute-on-chronic liver failure: a meta-analysis
Source: Virol J. 2015 Jun 11;12:87. doi: 10.1186/s12985-015-0313-5 (PMC4485863; doi:10.1186/s12985-015-0313-5)
Supplement: Additional file 2: Table S2. — Pooled unadjusted risk estimates of ACLF of specific mutation sites. [file 12985_2015_313_MOESM2_ESM.doc]

**Additional file 2: Table S2. Pooled unadjusted risk estimates of ACLF of specific mutation sites**

| mutation site | No.of included studies | No.of ACLF patients | No.of CHB patients | pooled OR | 95%CI | P value | Heterogeneity test | |
| --- | --- | --- | --- | --- | --- | --- | --- | --- |
|  |  |  |  |  |  |  | P | I2 |
| T1753V | 14 | 1337 | 2383 | 1.919 | 1.414 to 2.606 | <0.001 | 0.006 | 55.40% |
| A1762T | 16 | 1171 | 2226 | 2.685 | 2.264 to 3.185 | <0.001 | 0.164 | 25.70% |
| G1764A | 15 | 1149 | 2170 | 2.901 | 2.041 to 4.122 | <0.001 | 0.004 | 56.40% |
| A1762T/G1764A | 21 | 1313 | 2237 | 2.376 | 1.548 to 3.648 | <0.001 | <0.001 | 80.70% |
| C1766T | 10 | 1099 | 2015 | 1.849 | 1.403 to 2.437 | <0.001 | 0.849 | 0.00% |
| T1768A | 11 | 1163 | 2084 | 2.199 | 1.563 to 3.094 | <0.001 | 0.681 | 0.00% |
| A1846T | 11 | 556 | 921 | 3.163 | 2.157 to 4.639 | <0.001 | 0.037 | 48.20% |
| G1862T | 9 | 980 | 1616 | 1.802 | 0.737 to 4.404 | 0.196 | 0.001 | 73.10% |
| G1896A | 29 | 1949 | 3401 | 2.181 | 1.800 to 2.642 | <0.001 | 0.002 | 48.10% |
| G1899A | 16 | 1456 | 2507 | 3.525 | 2.882 to 4.312 | <0.001 | 0.307 | 12.80% |
| A1762T/G1764A/G1896A | 8 | 607 | 1069 | 1.575 | 1.172 to 2.116 | 0.003 | 0.073 | 46.10% |

NO.= number; ACLF= acute-on-chronic liver failure; CHB= chronic hepatitis B; OR= odds ratio.
